# Comparison of Intraoperative Ultrasound B-Mode and Strain Elastography for the Differentiation of Glioblastomas From Solitary Brain Metastases. An Automated Deep Learning Approach for Image Analysis

**DOI:** 10.3389/fonc.2020.590756

**Published:** 2021-02-02

**Authors:** Santiago Cepeda, Sergio García-García, Ignacio Arrese, Gabriel Fernández-Pérez, María Velasco-Casares, Manuel Fajardo-Puentes, Tomás Zamora, Rosario Sarabia

**Affiliations:** ^1^ Neurosurgery Department, University Hospital Río Hortega, Valladolid, Spain; ^2^ Radiology Department, University Hospital Río Hortega, Valladolid, Spain; ^3^ Pathology Department, University Hospital Río Hortega, Valladolid, Spain

**Keywords:** brain tumor, elastography, intraoperative ultrasound, deep learning, convolutional neural network

## Abstract

**Background:**

The differential diagnosis of glioblastomas (GBM) from solitary brain metastases (SBM) is essential because the surgical strategy varies according to the histopathological diagnosis. Intraoperative ultrasound elastography (IOUS-E) is a relatively novel technique implemented in the surgical management of brain tumors that provides additional information about the elasticity of tissues. This study compares the discriminative capacity of intraoperative ultrasound B-mode and strain elastography to differentiate GBM from SBM.

**Methods:**

We performed a retrospective analysis of patients who underwent craniotomy between March 2018 to June 2020 with glioblastoma (GBM) and solitary brain metastases (SBM) diagnoses. Cases with an intraoperative ultrasound study were included. Images were acquired before dural opening, first in B-mode, and then using the strain elastography module. After image pre-processing, an analysis based on deep learning was conducted using the open-source software Orange. We have trained an existing neural network to classify tumors into GBM and SBM *via* the transfer learning method using Inception V3. Then, logistic regression (LR) with LASSO (least absolute shrinkage and selection operator) regularization, support vector machine (SVM), random forest (RF), neural network (NN), and k-nearest neighbor (kNN) were used as classification algorithms. After the models’ training, ten-fold stratified cross-validation was performed. The models were evaluated using the area under the curve (AUC), classification accuracy, and precision.

**Results:**

A total of 36 patients were included in the analysis, 26 GBM and 10 SBM. Models were built using a total of 812 ultrasound images, 435 of B-mode, 265 (60.92%) corresponded to GBM and 170 (39.8%) to metastases. In addition, 377 elastograms, 232 (61.54%) GBM and 145 (38.46%) metastases were analyzed. For B-mode, AUC and accuracy values of the classification algorithms ranged from 0.790 to 0.943 and from 72 to 89%, respectively. For elastography, AUC and accuracy values ranged from 0.847 to 0.985 and from 79% to 95%, respectively.

**Conclusion:**

Automated processing of ultrasound images through deep learning can generate high-precision classification algorithms that differentiate glioblastomas from metastases using intraoperative ultrasound. The best performance regarding AUC was achieved by the elastography-based model supporting the additional diagnostic value that this technique provides.

## Introduction

Glioblastomas (GBM) represent approximately 40% to 50% of all malignant brain tumors (1). Brain metastases range from 9 to 17% in patients diagnosed with cancer; they may appear as single lesions and be the first manifestation of malignancy in 30%–50% of cases (2–4). The proper distinction of these tumors is essential because they have different treatments and prognosis.

The differential diagnosis of GBM and solitary brain metastases (SBM) can be difficult due to the similarity in conventional neuroimaging tests; both can present like single lesions, contrast-enhancing, with a cystic necrotic appearance and extensive involvement of perilesional white matter. Distinguishing them is particularly complicated when there is no evidence of a previous neoplasm. In these cases, more specific techniques such as PET (Positron Emission Tomography), specialized magnetic resonance imaging (MRI) sequences such as spectroscopy, diffusion/perfusion, and other forms of quantitative analysis can be used to clarify the origin of these lesions (5–18); however, in many centers, these techniques are not available, their acquisition and interpretation can sometimes be challenging and have a non-negligible margin of error.

Intraoperative diagnosis using frozen samples enables discriminating glial tumors from SBM obtaining a histopathological diagnosis after starting the tumor resection. Thus, it would be helpful to establish a surgical planning in the earliest stage of surgery. In the case of GBM, in our center, as in many others, the policy adopted is to try to carry out a supratotal resection whenever possible, taking into account the relationship with functional areas. In lobectomies, for example, resection includes non-enhancing tumor regions. This technique has been shown to improve the overall survival of these patients (19–25).

On the other hand, in metastases, resection is limited exclusively to the contrast-enhancing tumor component, because it is recognized that peritumoral MRI signal alterations are exclusively produced by vasogenic edema (26); therefore, there is still insufficient evidence to support supramarginal resections in these patients (27). Besides, in some cases, partial resections of brain metastases near functional areas might be indicated as a previous step to adjuvant therapies.

Intraoperative ultrasound is a low-cost, portable, fast technique that provides dynamic information in a real-time fashion. It has been widely used in brain tumor resection (28, 29); the simplicity of its application makes it a valuable intraoperative imaging option. Elastography is a relatively new technique in brain tumor pathology. Several publications highlight the importance of this technique because it provides better image contrast compared to B-mode and especially because it allows the characterization of elasticity patterns of the tumor and peritumoral regions, through which it is possible to differentiate between several histological types (30–37).

One of the disadvantages of medical imaging techniques is, of course, their interpretation. Regarding ultrasound, this technique presents challenges such as operator dependency, noise and artifacts. Deep learning is a branch of machine learning that has emerged to improve classification tasks using visual computer systems. The basic idea is that medical images have much more information than the human eye can process and distinguish. Deep learning involves the computation of hierarchical features or representations of a sample, in which high-level abstract features are defined by combining them with other low-level features (38). A deep learning approach based on convolutional neural networks (CNN) is getting attention in the medical image field. Artificial neural networks use a multi-step process that automatically learns features from an image and then extracts them to perform a classification task using an algorithm. CNN’s are designed to automatically and adaptively learn features from data through backpropagation using multi-block reconstruction called convolution layers, pooling layers, and fully connected layers (39). Transfer learning is a technique that allows the use of a pre-trained CNN model. It has been previously used in oncological classification tasks with high accuracy. Several studies have demonstrated the ability of transfer learning to work with small datasets using minimal image pre-processing (40–44).

The objective of our work is to use intraoperative ultrasound images and a CNN-based deep learning model in order to differentiate GBM from SBM. We seek to assess the intraoperative ultrasound accuracy on this task while comparing B-mode against an emerging technique such as elastography.

## Material and Methods

### Patient Selection

A retrospective analysis of patients diagnosed with supratentorial tumors who underwent surgery by craniotomy between March 2018 and June 2020 was performed. Those cases with histopathological diagnosis of glioblastomas and metastases that had an intraoperative ultrasound study were included. Approval was obtained from the ethics committee of our center as well as patient’s informed consent in all cases. Clinical, radiological and histopathological variables were collected.

### Acquisition and Pre-Processing of Ultrasound Images

**Figure 1:**
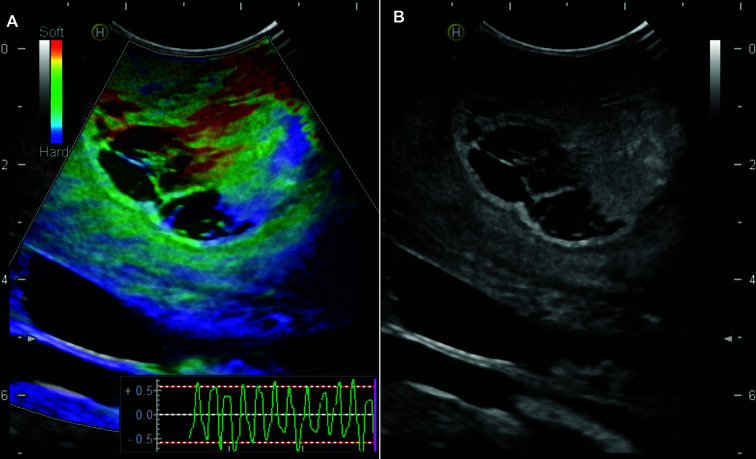
Example of intraoperative ultrasound images. 65-year-old man with a right frontal glioblastoma. **(A)** Elastogram showing the difference in consistency between the tumor and the peritumoral region (green - red) from the rest of the healthy parenchyma (blue). In the right-lower part of the image, a graphic representation of the external compression waves is observed. **(B)** Simultaneous image in B-mode.

For the intraoperative ultrasound study, we used the Hitachi Noblus with a C42 convex probe, 8-10 MHz frequency range, 20 mm scan width radius and 80° scan angle of field of view. The images were acquired after the craniotomy and before the dural opening. The probe is placed perpendicular to the dura; manual compressions were performed maintaining a constant rhythm and intensity. More details of the elastogram acquisition technique are described in a previous publication by our group (34). The ultrasound machine generates a real-time color map called elastogram simultaneously with B-mode. **Figure 1**. The color scale represents the tissue’s elasticity/consistency, with tones ranging from red (soft) to blue (hard). Elastograms and B-mode images attempted to cover the highest possible tumor volume and peritumoral areas with evident echogenicity changes. Several slices in different planes were acquired. The images were stored in DICOM format for offline processing.

**Figure 2:**
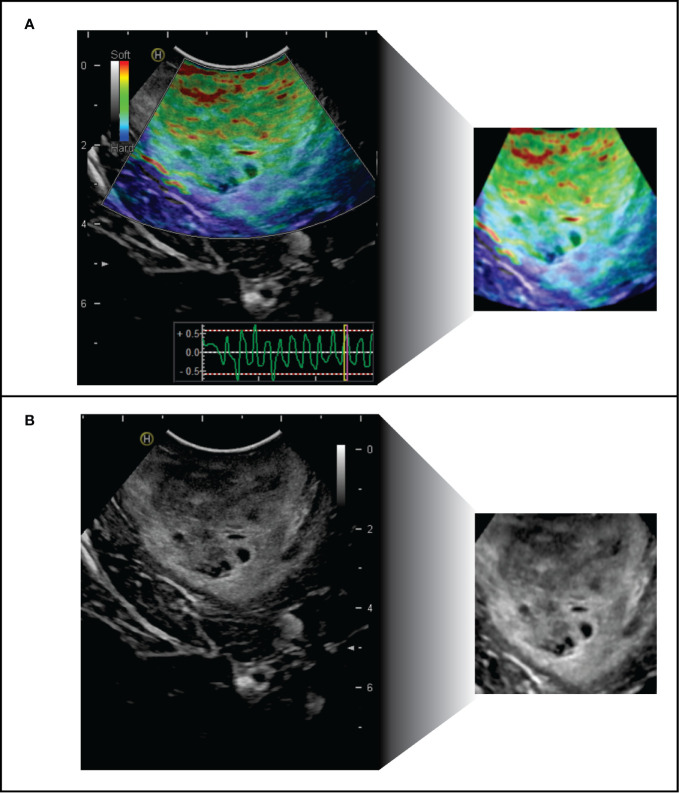
Intraoperative ultrasound images pre-processing. Left: original images of **(A)** elastogram and **(B)** B-mode. Right: Final image available for automatic analysis.

The open-source software ImageJ version 1.50i (National Institutes of Health, Maryland, United States) was used to pre-process the ultrasound images. The first step was to convert DICOM files to 8-bit TIFF format. For the B-mode images, the tumor and peritumoral area were cropped, removing possible small peripheral artifacts and dark areas. Images with significant artifacts or with unrecognizable areas on elastography were excluded. In the case of elastograms, the area of the elastogram was cut out by removing the periphery that corresponded to zones in B-mode. A rescaling was then performed at 299 x 299 pixels; the intensities were normalized, despeckling, and smoothing by Gaussian blur filter was carried out; thus, we obtained images with similar intensities and standardized size for the analysis. **Figure 2**.

### Analysis Using Deep CNN *via* Transfer Learning

**Figure 3:**
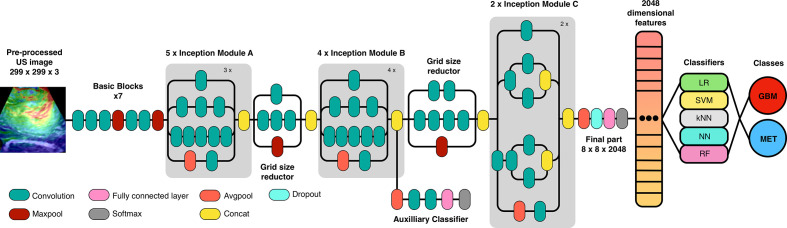
Schematic representation of Inception v3 architecture (adapted from GoogLeNet) and the workflow used in the transfer learning process *via* convolutional neural network and classification algorithms.

For the generation of an image classification system, the open-source software Orange version 3.26 (University of Ljubljana, Slovenia) was used. The software has a user-friendly interface based on a work panel and the use of widgets. **Supplementary Figure 1**. After importing the images into the workspace, the first step consisted in the process called embedding. Using preprocessed ultrasound images, we have trained an existing neural network to classify tumors into GBM and SBM. Thus, we have used a transfer learning method applying Inception V3, one of the most popular image recognition models that have been previously adapted to the analysis of medical images with excellent results (45–47). Inception V3 is a 48-layer convoluted neural network trained in 1.2 million images from the ImageNet repository (48); each image in the ImageNet Large Scale Visual Recognition Challenge repository belongs to one of the 1000 defined classes. Inception architecture is schematically summarized in **Figure 3**. Embedding process relies on the penultimate of these deep networks, where transfer learning is achieved by encoding images with characteristics of this layer, so each image is embedded in a 2048-element vector, followed by classic machine learning algorithms.

### Hierarchical Clustering

In the first phase of machine learning, and without previously establishing categories or classes, the distances of the vector representations of all the images were calculated using the cosine as a distance metric. From the distance matrix, a hierarchical classification was made into groups called clusters. The software automatically detects related elements in search of patterns. To determine the elements included in each cluster, an analysis of the GBM and SBM categories’ distribution within each subgroup was performed, both in B-mode and elastography. These groups are represented graphically through the development of a dendrogram.

### Classifiers and Model Validation

In order to develop a prediction model, the following classification algorithms were used: logistic regression (LR) applying LASSO (least absolute shrinkage and selection operator) regularization, Neural Network (NN), Random Forest (RF), Support Vector Machine (SVM), and k-Nearest Neighbor. Model validation was performed using a ten-fold stratified cross-validation. Most of the sample was used in the construction or learning process of the model, leaving a portion of the sample for the validation of its predictions, the stratification maintains the proportion of both categories, namely GBM and SBM, this step is repeated several times guaranteeing that the cases were distributed randomly as part of the training and test group. For this reason, cross-validation has proved to be superior to the simple split random sampling. The models were evaluated using the AUC (area under the curve)/ROC (receiver operating characteristics), classification accuracy (CA) and precision scores. Furthermore, confusion matrices were developed to determine the correct and misclassified cases for each algorithm.

### Comparison of the Automatic Model Versus Experienced Human Observers

After establishing the best classification algorithm, a training set made up of 70% of the sample was randomly selected, a predictive model was built and then was validated in the test-data set, 30% remaining of the sample, keeping the proportion of each of the classes. Using the same test-data set, two expert observers analyzed the images, classifying them as GBM and SBM according to their judgment. One of them is a senior neuroradiologist with ten years of experience, and the second observer is a neurosurgeon with thirty years of experience and knowledge about intraoperative ultrasound images. Both observers were blinded to the definitive histopathological diagnosis. Their results were compared with the automatic algorithm.

## Results

**Figure 4:**
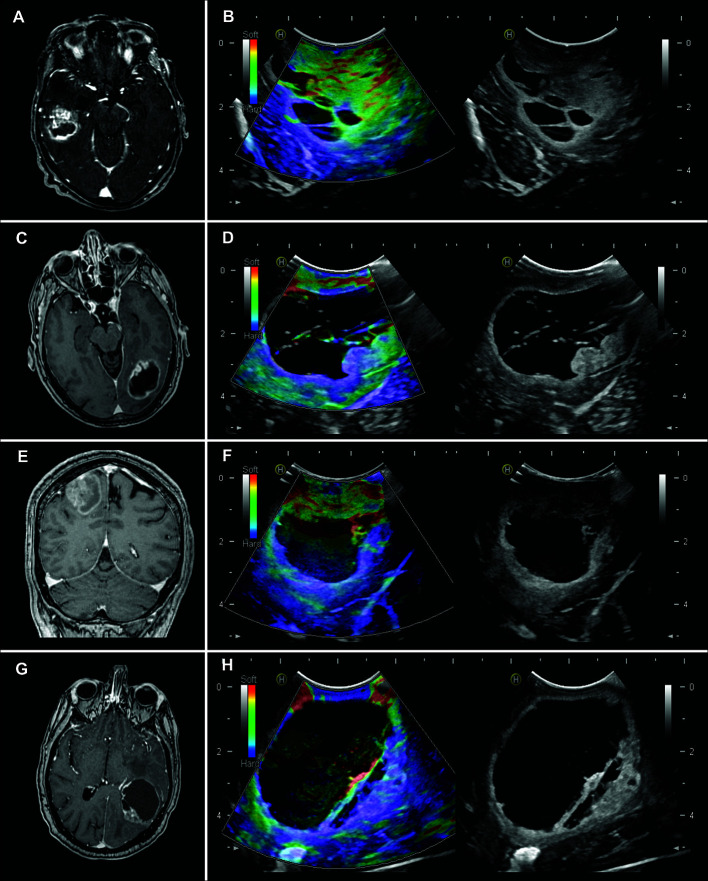
Illustrative cases of the use of intraoperative ultrasound. **(A)** Axial T1 weighted post-contrast (T1WC) image of a 50-year-old man with a right temporal glioblastoma. **(B)** Elastogram (left) and B-mode (right). It is a soft tumor with small cystic regions and a peritumoral region of low stiffness compared to the healthy parenchyma. **(C)** Axial T1WC image of a 70-year-old woman with a left occipital glioblastoma. **(D)** The elastogram shows a cystic/necrotic lesion with a nodular component of intermediate consistency and a relatively soft peritumoral region. **(E)** Coronal T1WC image of a 45-year-old man with a right parietal lung metastasis. **(F)** The elastography image shows a solid/cystic lesion with a soft nodular component and a stiffer peritumoral region. **(G)** Axial T1WC image of a 52-year-old man with no history of systemic cancer with a left parieto-occipital metastasis. **(H)** The elastogram shows a large cystic lesion with a small hard region and a peritumoral region of similar consistency.

Thirty-six patients were included during the study period. Twenty-six cases corresponded to GBM and 10 to metastases. The histological diagnoses, radiological and demographic characteristics, are summarized in **Table 1**. Illustrative cases and their appearance on MRI and intraoperative ultrasound images are shown in **Figure 4**.

**Table 1:** Patient characteristics.

Variable	n
Age	
Sex Female Male	64.58 ± 8.76 10 (27.8%) 26 (72.2%)
Preoperative KPS	77.78 ± 9.88
Histopathology Glioblastoma Metastases Lung Breast Ovarian Colorectal Prostate	26 (72.2%) 10 (27.8%) 6 (60%) 1 (10%) 1 (10%) 1 (10%) 1 (10%)
Tumor location Frontal Parietal Temporal Occipital	16 (44.4%) 8 (22.2%) 8 (22.2%) 4 (11.1%)
Initial volume (cm^3^)	25.31 ± 24.27

Values are expressed as the mean ± standard deviation or as the frequency (%). KPS, Karnofsky Performance Score.

**Figure 5:**
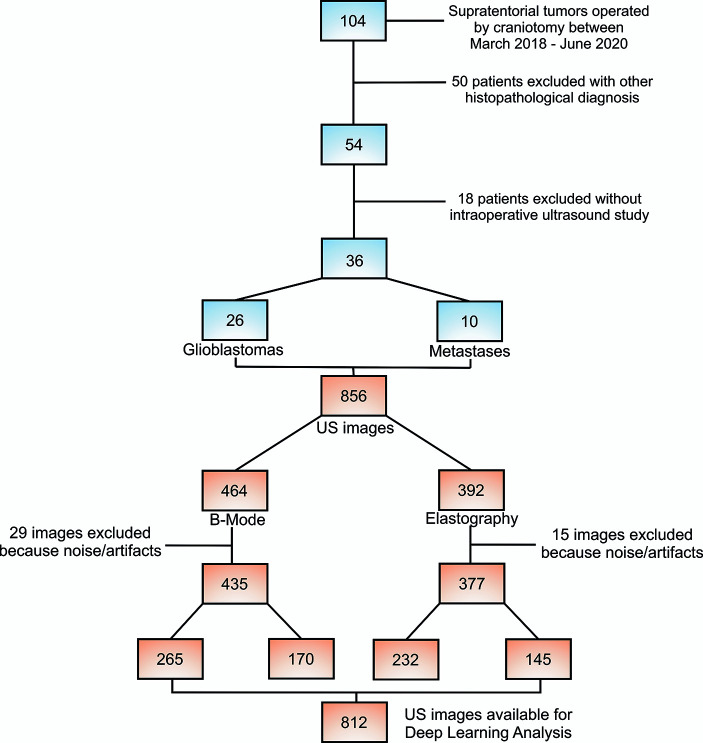
Flow chart of patient and ultrasound image selection process.

Models were built using a total of 812 ultrasound images, 435 of B-mode, 265 (60.92%) corresponded to GBM and 170 (39.8%) to metastases. In addition, 377 elastograms, 232 (61.54%) GBM and 145 (38.46%) metastases were analyzed. **Figure 5**. The average of B-mode images was twelve images per patient, while for elastography, an average of eleven images was analyzed for each case. The difference in the number of images between the two modalities is because several images were discarded due to their low quality or to the presence of noise/artifacts.

**Figure 6:**
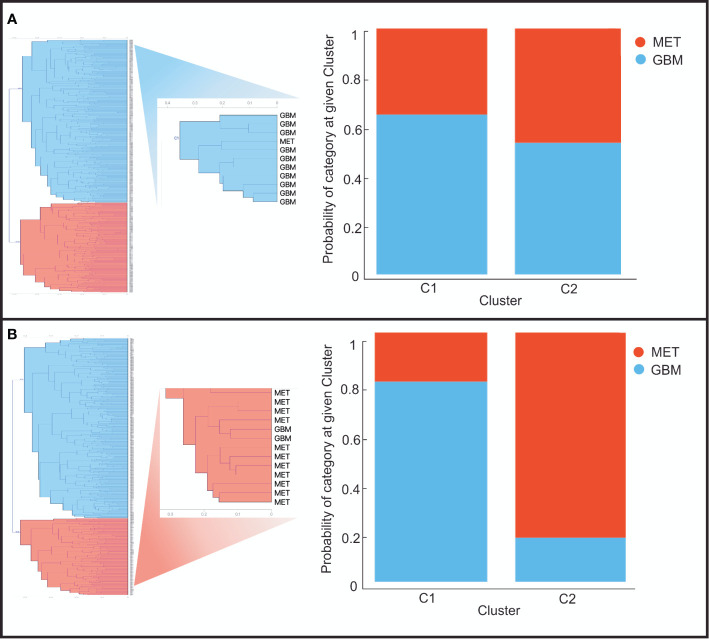
Graphical representation of the clusters generated from the distance matrices after analyzing images in **(A)** B-mode and **(B)** Elastography. Left: dendrograms of the top two clusters. Right: Bar graph of the probabilities of being assigned to each cluster of glioblastomas (blue) and metastases (red).

By hierarchical clustering, two main groups of images were identified. For B-mode, the first cluster included 65% of GBM images and the second cluster 46.45% metastasis. For elastography, the first cluster contained 80.3% GBM and the second cluster 82.3% metastases. The dendrogram of **Figure 6** graphically demonstrated the distribution and results of the classification.

**Figure 7:**
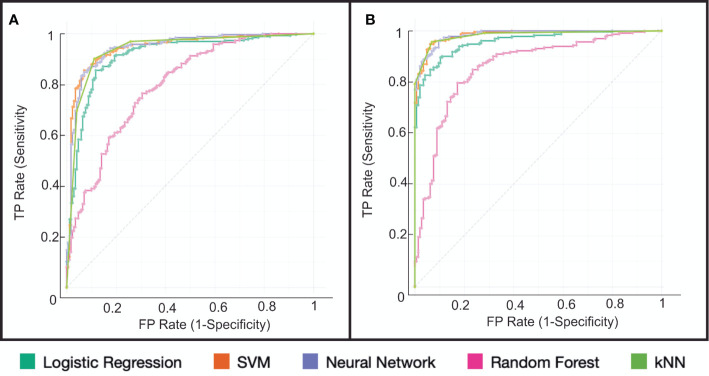
Representation of classifier performance using the ROC (Receiver Operating Characteristics) curve for **(A)** B-mode and **(B)** Elastography. The best results were obtained by the Support Vector Machine (SVM) and K-Nearest Neighbor (k-NN) algorithms.

The performance of the classification algorithms was represented graphically using the ROC (Receiver Operating Characteristics) curves. **Figure 7**. For B-mode, the classification algorithms’ AUC and accuracy values ranged from 0.790 to 0.943 and from 72 to 89%, respectively. **Table 2**. Elastography-based model demonstrated the best performance since AUC and accuracy values ranged from 0.847 to 0.985 and 79 to 95%. **Table 3** and **Supplementary Figure 2**.

**Table 2:** Diagnostic performance of classification algorithms based on Ultrasound B-mode images.

Classifier	AUC	CA	F1-Score	Precision	Recall
kNN	0.938	0.897	0.897	0.898	0.897
Logistic Regression	0.915	0.871	0.871	0.871	0.871
Neural Network	0.945	0.876	0.877	0.879	0.876
Random Forest	0.791	0.749	0.724	0.779	0.749
SVM	0.944	0.887	0.887	0.887	0.887

AUC, Area Under the Curve; CA, Classification Accuracy; kNN, k-Nearest Neighbor; SVM, Support Vector Machine.

**Table 3:** Diagnostic performance of classification algorithms based on Ultrasound Elastography images.

Classifier	AUC	CA	F1-Score	Precision	Recall
kNN	0.983	0.947	0.947	0.947	0.947
Logistic Regression	0.960	0.889	0.888	0.888	0.889
Neural Network	0.985	0.918	0.918	0.922	0.918
Random Forest	0.861	0.796	0.786	0.803	0.796
SVM	0.985	0.941	0.941	0.942	0.942

AUC, Area Under the Curve; CA, Classification Accuracy; kNN, k-Nearest Neighbor; SVM, Support Vector Machine.

After the random selection of cases, the human observers’ results versus the automatic selection algorithm are summarized in **Table 4**. The accuracy achieved by the experienced observers was up to 61% in the case of B-mode and 68% for elastography. For the CNN-based automatic system, the accuracy was 88% in B-mode and 93% in elastography.

**Table 4:** Comparison between convolutional neural network (CNN)-SVM model performance and the two expert observers.

Ultrasound Modality	Classifier	AUC	CA	Precision
**B-mode**	SVM	0.937	0.877	0.883
	Observer 1	0.573	0.608	0.659
	Observer 2	0.545	0.569	0.642
**Elastography**	SVM	0.976	0.929	0.930
	Observer 1	0.622	0.681	0.693
	Observer 2	0.587	0.612	0.686

AUC, Area Under the Curve; CA, Classification Accuracy; SVM, Support Vector Machine.

## Discussion

In the present study, we have developed a highly accurate classification system that allows GBM to be differentiated from SBM using automatic intraoperative ultrasound image processing through convolutional neural networks. Furthermore, elastography showed slightly better performance for the classification of these tumors compared to the B-mode.

Among the strengths of our work, we can mention that it is the first time that intraoperative ultrasound B-mode and elastography are applied to discriminate glioblastomas from metastases. Besides, our study follows a cutting-edge methodology, in which deep learning techniques are applied in the analysis of ultrasound images, the combination of these two technologies in brain tumor pathology has no previous references in the literature.

We are aware of our limitations, it is worth mentioning that the sample size from which we started is relatively small. This aspect can cause an overfitting problem and the creation of an over-optimistic predictive model. Our strategy to overcome this issue was to use all the images available in each case, including different sections and projections of each tumor. We reached a sample size and a balance of classes enough to carry out an analysis based on artificial intelligence techniques.

On the other hand, we recognize the limitations that elastography holds, such as the variability of elasticity threshold values and the absence of an image quality control; also, they often contain irrelevant patterns that can difficult both handcrafted feature extraction and DL methods such as CNN.

Deep learning, a branch of machine learning, can automatically process and learn mid-level and high-level abstract characteristics acquired from raw data, in this case, ultrasound images. Still, tumor classification into subtypes is difficult due to variations in shape, size, intensities, and because different histological types can show similar patterns.

The image acquisition and processing methodology are rigorous and clear in every step. Strain type elastography is a technique used in previous studies, and with promising results regarding its application in the resection of brain tumors (34, 36). Pre-processing ultrasound images is a fundamental step, which has been performed with the highest reliability, applying a user-friendly open-source software that performs robust analysis without adding complexity (49, 50).

The analysis through deep learning has been demonstrated to be superior in image recognition compared to conventional radiological techniques and handcrafted radiomics (51, 52). The methods applied in our study, suppress some cumbersome steps such as tumor segmentation, which implies a significant limitation in this type of work and may bias the selection of variables of interest, such as texture features. The difference is that CNN, through transfer learning, takes advantage of a previously trained network of proven validity, to generate classification systems that automatically and without human intervention can distinguish between one class or another, in our case, GBM from SBM. A disadvantage of these systems is the inability to know which characteristics the software has used to generate its predictions, sometimes compared to a “black box” (53). Although feature selection techniques could be applied after converting images to vector representations, these techniques are still not validated. Using DL models, we can lose interpretability in exchange for gaining more robust and generalizable prediction systems based on much more complex characteristics.

A comparison has been conducted between the classification algorithms and experienced human observers to discriminate these tumor types using ultrasound images in our study. According to our results, the DL based model seems to be more precise and accurate to differentiate one tumor type from another. These findings make it necessary to improve our knowledge about how artificial intelligence works, only in this way, these new technological resources will serve as support tools in neurosurgical and radiological fields.

In our study, the best performance regarding AUC and accuracy was achieved by the elastography-based model compared to B-mode in the classification task of SBM and GBM. One possible explanation for this advantage could be the better contrast offered by the color images of the elastograms, as previously published (33). Furthermore, we believe that one of the fundamental differences between the two tumor types is the correlation between the peritumoral regions’ histology and their radiological appearance. Although this correlation has not been proven, previous studies indicate that the elasticity patterns differ in gliomas and metastases (34, 54). These differences do not seem to be captured with the B-mode; thus, the peritumoral areas’ elastographic pattern could differentiate both histological types through automatic analysis of this imaging modality. It is worth mentioning that the elastograms are produced in an RGB (red-green-blue) image format. Therefore, the elastogram results from the superposition of the B-mode image and the colorimetric scale of the tissues’ elasticity. Our study has been carried out based on the original image produced by the ultrasound machine because we wanted to use the same pictures in the classification task by human observers. Another alternative for future studies could be to perform an image decomposition in HSB (hue-saturation-brightness) format and then extract the hue component.

Regarding the differentiation of GBM from SBM, we know that there are multiple radiological techniques available for pre-operative or non-invasive applications (5, 8–11, 13, 15–17, 55); besides, intraoperative histopathological techniques are currently the reference parameter for decision-making (56). Our study does not intend to make a comparison with the techniques mentioned above but to demonstrate, on the one hand, the high value that ultrasound and especially elastography owns in the study of brain tumors, and on the other hand, highlight that automatic image processing is a highly reliable technique. Therefore, we believe that it is essential to develop automatic ultrasound image analysis methods to increase the precision in the diagnosis, evaluation, and interventionism based on this technique.

Our work demonstrates that automated processing of ultrasound images through deep learning can generate high-precision classification algorithms that differentiate glioblastomas from metastases using intraoperative ultrasound. The best performance regarding AUC and accuracy was achieved by the elastography-based model, supporting the additional value that this technique provides by analyzing brain tumor elasticity. With our results, the next step will be to obtain real-time feedback based on intraoperative image analysis, allowing the surgeon to adapt the surgical strategy and even guide tumor resection.

## Data Availability Statement

The raw data supporting the conclusions of this article will be made available by the authors, without undue reservation.

## Ethics Statement

The studies involving human participants were reviewed and approved by University Hospital Río Hortega Ethics Committee. The patients/participants provided their written informed consent to participate in this study.

## Author Contributions

Conception and design: SC and RS. Material preparation, data collection, and analysis were performed by SC, SG-G, MV-C, GF-P, IA, MF-P, and TZ. The first draft of the manuscript was written by SC and all authors commented on previous versions of the manuscript. All authors contributed to the article and approved the submitted version.

## Conflict of Interest

The authors declare that the research was conducted in the absence of any commercial or financial relationships that could be construed as a potential conflict of interest.

## Supplementary Material

Supplementary Figure 1 Orange visual environment and the workflow used in the construction of the predictive model.

Supplementary Figure 2 Confusion matrices generated by the different classification algorithms based on (left) B-Mode and (right) Elastography. **(A)** k-Nearest Neighbor; **(B)** Logistic Regression; **(C)** Neural Network; **(D)** Random Forest and **(E)** Support Vector Machine. The number of instances correctly (purple) and misclassified (pink) are shown.
