# Supplementary figures and images for: Comparison of Intraoperative Ultrasound B-Mode and Strain Elastography for the Differentiation of Glioblastomas From Solitary Brain Metastases. An Automated Deep Learning Approach for Image Analysis
Source: Front Oncol. 2021 Feb 2;10:590756. doi: 10.3389/fonc.2020.590756 (PMC7884775; doi:10.3389/fonc.2020.590756)

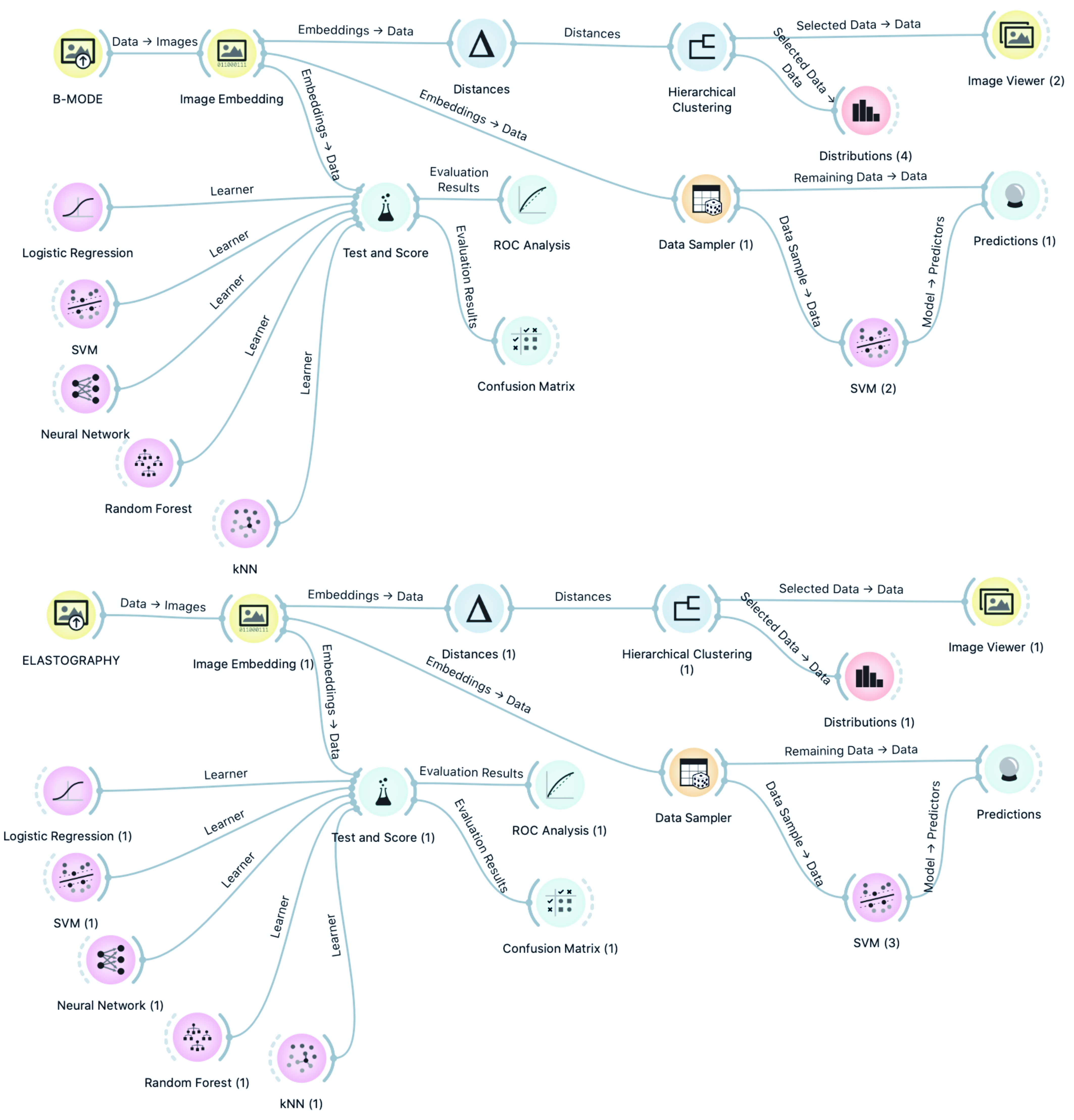

Supplement: Supplementary Figure 1 — Orange visual environment and the workflow used in the construction of the predictive model. [file Image_1.jpeg]

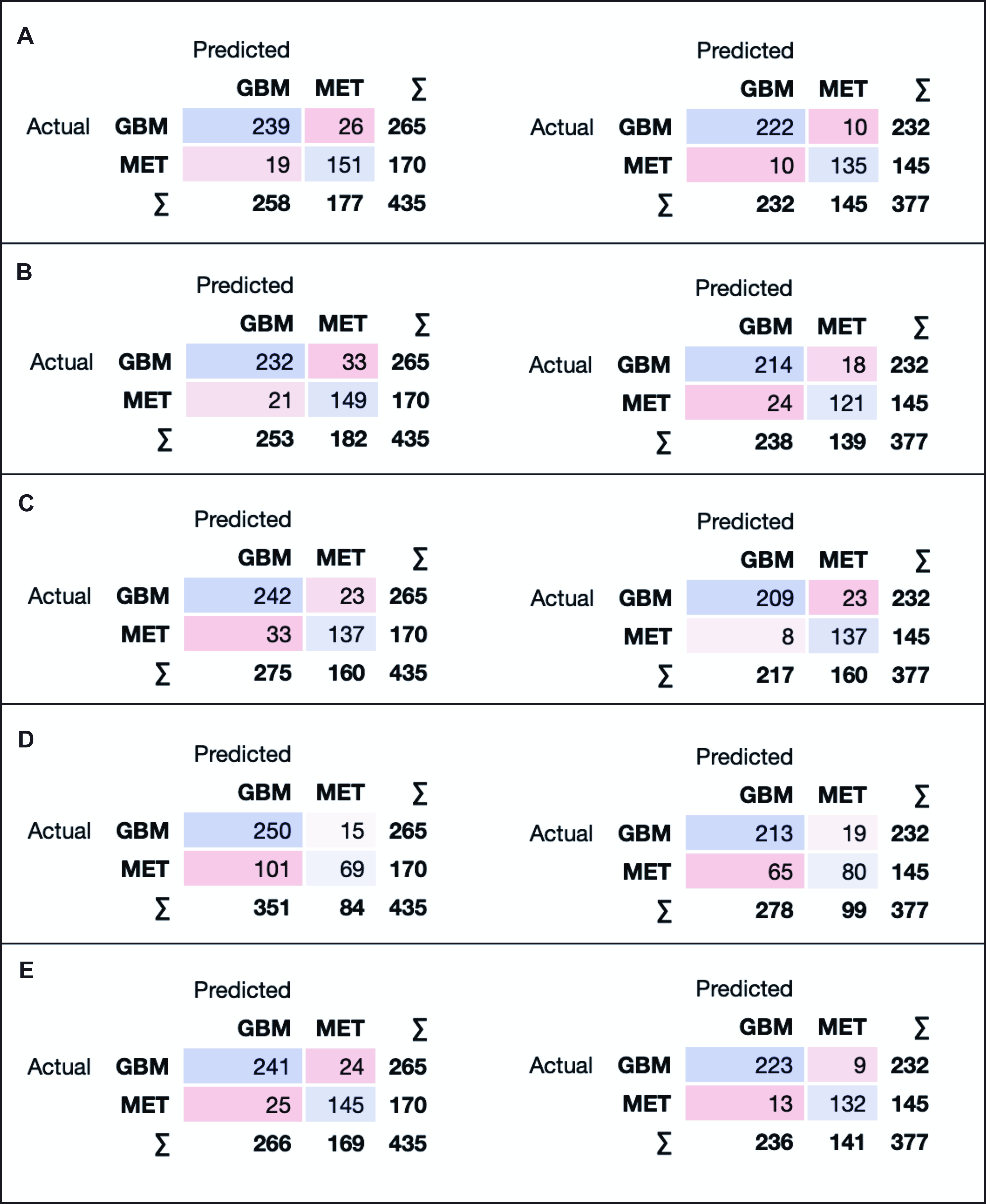

Supplement: Supplementary Figure 2 — Confusion matrices generated by the different classification algorithms based on (left) B-Mode and (right) Elastography. (A) k-Nearest Neighbor; (B) Logistic Regression; (C) Neural Network; (D) Random Forest and (E) Support Vector Machine. The number of instances correctly (purple) and misclassified (pink) are shown. [file Image_2.jpeg]
